# Association of heavy menstrual bleeding with cardiovascular disease in US female hospitalizations

**DOI:** 10.1186/s12916-024-03426-8

**Published:** 2024-05-23

**Authors:** Pallavi Dubey, Sireesha Reddy, Vishwajeet Singh, Abdelrehman Yousif, Alok Kumar Dwivedi

**Affiliations:** 1 Department of Obstetrics and Gynecology, Paul L. Foster School of Medicine, Texas Tech University Health Sciences Center El Paso, El Paso, TX USA; 2 Biostatistics and Epidemiology Consulting Lab, Office of Research, Texas Tech University Health Sciences Center El Paso, El Paso, TX USA; 3 Division of Biostatistics & Epidemiology, Department of Molecular and Translational Medicine, Paul L. Foster School of Medicine, Texas Tech University Health Sciences Center El Paso, El Paso, TX USA

**Keywords:** Menorrhagia, Heavy menstrual bleeding, Cardiovascular disease, Irregular menstruation, Obesity, Metabolic syndrome

## Abstract

**Background:**

Heavy menstrual bleeding (HMB) is a common menstrual disorder associated with multiple risk factors of cardiovascular disease (CVD) in women. In addition, HMB is often present with irregular menstruation (IM) which is a risk factor for CVD outcomes. However, the relationship between HMB and CVD outcomes is unexplored in the presence or absence of IM. We determined the association of HMB with multiple CVD outcomes using a nationally representative sample of female hospitalizations in the US.

**Methods:**

All hospitalizations of females with HMB diagnosis and normal menstrual cycles from ages of 18 to 70 years were extracted from the National Inpatient Sample Database, 2017. The HMB was defined using the International Classification of Diseases (ICD)-10 for excessive and frequent menstruation bleeding and included any current or history of HMB diagnosis. Outcomes including major adverse cardiovascular events (MACE), coronary heart disease (CHD), stroke, heart failure (HF), atrial fibrillation (AF) or arrhythmia, myocardial infarction (MI), and diabetes (DM) were defined using ICD-10 codes. Adjusted logistic regression and prosperity scores-matched logistic regression analyses were conducted to summarize adjusted associations with an odds ratio (OR) and a 95% confidence interval (CI).

**Results:**

Among 2,430,851 hospitalizations, HMB was observed in 7762 (0.68%) females with age ≤ 40 years and 11,164 (0.86%) females with age > 40 years. Among hospitalizations with age ≤ 40 years, HMB was significantly associated with increased odds of CVD outcomes including MACE (OR = 1.61; 95% CI: 1.25, 2.08), CHD (OR = 1.72; 95% CI: 1.10, 2.71), stroke (OR = 1.95; 95% CI: 1.12, 3.40), HF (OR = 1.53; 95% CI: 1.15, 2.03), and AF/arrhythmia (OR = 1.84; 95% CI: 1.34, 2.54). These associations were confirmed in multiple sensitivity analyses. In contrast, HMB was not robustly associated with CVD events among hospitalizations of women with age > 40 years. HMB without IM was strongly associated with DM, HF, AF, and MACE outcomes while HMB with IM was strongly associated with CHD and AF outcomes in hospitalizations of young women.

**Conclusions:**

HMB is associated with CVD events among US hospitalizations of young women. A routine investigation and screening of menstrual disorders, especially HMB, is useful for CVD risk stratification and management in young women.

**Supplementary Information:**

The online version contains supplementary material available at 10.1186/s12916-024-03426-8.

## Background

Cardiovascular disease (CVD) is the leading cause of mortality with a global burden of approximately 19 million deaths in 2020, accounting for about 32% of all global deaths [1]. Considering sex differences and the increasing prevalence of metabolic syndrome (MS) and CVD, particularly in women, it becomes critical to identify modifiable risk factors for CVD risk prevention in women [2–4]. Menstrual disorders refer to a wide range of conditions that impact a woman's menstrual cycle, affecting several factors such as regularity, duration, severity, or other associated symptoms yielding severe complications and adverse health outcomes [5, 6]. Heavy menstrual bleeding (HMB), a type of menstrual disorder, involves excessive menstrual blood loss of greater than 80 mL per menstrual period or clinically excessive bleeding that affects the physical, emotional, and social distress, and quality of life of affected females [7]. HMB is one of the prevalent menstrual disorders of reproductive-aged women affecting 9% to 52% of women depending on objective and subjective assessments of HMB [8]. HMB is also referred to as menorrhagia and is sometimes interchanged with abnormal uterine bleeding as it often occurs due to uterine/endometrial, hormonal, or coagulation-related conditions [9]. Approximately one-third of gynecological visits in the US are due to HMB and it represents a heavy economic burden on patients for treatment costs and productivity loss [8, 10]. It is typically associated with anemia, fatigue, headache, and pain [11]. Its association with iron deficiency anemia can impair oxygen transport and interfere with cardiac function [12]. In addition, hormonal-related changes and other associated morbidities of HMB may adversely affect cardiometabolic health leading to CVD outcomes [11, 13].

HMB has appeared to be associated with multiple risk factors including obesity and hypertension that may increase the risk of heart disease[4, 11, 14–16]. Moreover, HMB is associated with secondary polycystic ovary syndrome (PCOS), insulin resistance, and inflammation indicating that HMB can be a risk factor for CVD outcomes [15, 17, 18]. However, a direct association between HMB and CVD outcomes has not been evaluated. Since irregular menstruation (IM) is considered the prominent feature of PCOS[19, 20], several studies demonstrated a positive association of IM with CVD outcomes and mortality [21, 22]. However, a substantial proportion of HMB women do not have an IM comorbidity. It is unclear whether HMB without IM is robustly associated with CVD outcomes to a greater extent than HMB with IM. Therefore, we sought to determine the association of HMB with or without IM with multiple CVD outcomes using a nationally representative sample of hospitalizations in the US.

## Methods

### Study population

We conducted a retrospective cross-sectional study using the National Inpatient Sample (NIS) Database from 2017. The NIS is a publicly available all-payer inpatient healthcare database designed to produce US regional and national estimates of inpatient outcomes, resources, and costs. The NIS data collection and procedures followed Helsinki’s declaration of ethical standards. The NIS, 2017 includes de-identified datasets from the International Classification of Diseases, 10th Revision (ICD-10) codes from participating hospitals and hence, does not require participant consent or institutional review board approval. In our study, we included all hospitalizations of women with HMB regardless of IM status and no menstrual disorders of ages between 18–70 years. Only limited cases had HMB in females aged > 70 years and thus excluded from the study. Since the pathophysiology of other menstrual disorders is different from HMB, we excluded hospitalizations with hematocolpos, amenorrhea, excessive menstruation at puberty, ovulation bleeding, only IM, and dysmenorrhea.

### Outcomes

The study outcomes were major adverse cardiovascular events (MACE), coronary heart disease (CHD), heart failure (HF), stroke /cardiovascular accident (CVA), myocardial infarction (MI), atrial fibrillation (AF)/arrhythmia, and diabetes (DM). Due to limited CVD mortality and no coronary revascularization, MACE was defined as the presence of any events related to stroke, MI, and HF [20]. These outcomes were defined using the ICD-10 codes provided in our publication [20].

### Exposure

The primary exposure variable was HMB. We included women with excessive and frequent menstruation with regular cycles (using ICD-10: N920) considered as HMB without IM. We also included women with excessive and frequent menstruation without regular cycles (using ICD-10: N921) considered as HMB with IM. These ICD codes account for all hospitalizations with a current or history of HMB. We also included all eligible hospitalized women without menstrual disorders (normal menstrual cycle-NMC) for comparison.

### Covariates

We included an extensive set of covariates including demographics, risk factors, medical conditions, and treatments known to serve as confounders for the association between HMB and CVD outcomes. The covariates considered in this study were age (years), race/ethnicity (white, black, Hispanic, and other/missing), household (HH) income quartile (1st, 2nd, 3rd, 4th, and missing), primary payer (Medicare, Medicaid, private insurance, self-pay, no charge, and others/missing), smoking status (no and yes), alcohol (no and yes), obesity (no and yes), and MS (no and yes), contraceptive or hormonal use, PCOS, anemia, anticoagulant, non-steroidal anti-inflammatory drugs (NSAIDs), and leiomyoma uterus. MS was defined as the presence of at least three conditions among fasting glucose, low high-density lipoprotein, high blood pressure, high triglyceride, and obesity [20]. For validation analyses, we additionally extracted information on insulin use, infertility for pregnancy status, and inflammatory bowel disease (IBD). All the demographic covariates were extracted from the database as recorded in the NIS. However, the medical conditions were extracted using the appropriate ICD-10 codes.

### Statistical analysis

All the statistical analyses were carried out by applying the appropriate sampling weight following the Healthcare Cost and Utilization Project (HCUP)-NIS protocol [20]. Data were summarized with appropriate descriptive statistics including mean with standard deviation or frequencies with percentages. The association between HMB and each CVD outcome was evaluated using survey-weighted logistic regression analyses. We explored all possible interactions with HMB by adding a multiplicative term with HMB in regression analyses. Since age was a modifier for the association between HMB and most CVD outcomes, the results were presented for ages ≤ 40 years and > 40 years, separately. A priori, all the considered covariates were included in the analyses as per the study objective in the propensity scores-matched analysis. A logistic regression model was developed to generate propensity scores for HMB by considering all covariates. We used a caliper of 0.001 to identify matched samples of propensity scores of HMB and NMC groups. A survey-weighted logistic regression was then performed for each CVD outcome in propensity scores-matched samples after additionally adjusting for age. We further validated results for hospitalized women aged ≤ 40 years by conducting survey-weighted logistic regression models (a) model 1 included all primary covariates, i.e., age, race/ethnicity, household income quartile, primary payer, smoking, alcohol, contraceptive/hormone use, MS, NSAIDs, and leiomyoma uterus, and (b) model 2 included model 1 covariates plus hypertension, high triglyceride, fasting glucose, low high-density lipoprotein, insulin use, obesity, DM, IBD, infertility, and anemia. The results of logistic regression were summarized with odds ratio (OR), corresponding 95% confidence interval (CI), and p-value. Since the prevalence ratio (PR) is an appropriate effect size measure in a cross-sectional study compared to the OR, the primary model associations were also validated using modified Poisson regression models to estimate the PR, with a 95% CI [23]. We performed various sensitivity analyses to confirm our study findings. The findings obtained from primary models were validated after excluding (a) PCOS, (b) leiomyoma uterus, (c) congenital heart disease, (d) anticoagulation use, (e) NSAID use, and (f) lifestyle factors (smoking/alcohol use and obesity). The adjusted direct and indirect associations of HMB with MACE outcome through mediators including MS, obesity, hypertension, DM, and anemia were evaluated using the decomposition method for logistic regression [24]. The association of each covariate with MACE outcome determined using a survey-weighted logistic regression was also summarized with OR, 95%CI, and p-value. We also evaluated the association between HMB with or without IM and each CVD outcome using survey-weighted logistic regression analyses. Among hospitalizations of women aged > 40 years, we conducted a stratified analysis by age group (41–55 vs. 56–70 years) to evaluate the impact of menopause transition using a propensity scores-matched analysis followed by survey-weighted logistic regression analyses. STATA 17.0 version was used for the data management and statistical analyses. A p-value less than 5% was considered a statistically significant result. We followed statistical analysis and methods in biomedical research (SAMBR) checklists and conducted statistical analysis as per the guidance resource [25].

## Results

### Subject characteristics

A total of 2,430,851 records of hospitalized women were found to be eligible for statistical analysis. The mean (standard deviation) age of the patient hospitalizations was 44.4 (16.0) years. Over half of the hospitalizations were non-Hispanic white followed by non-Hispanic black (17.4%) and Hispanic (13.4%). One-third of the hospitalized women (31.3%) had household income in the first quartile and primary payers were mostly private (39.4%), Medicaid (29.6%), or Medicare (23.6%). A limited proportion of hospitalized women (0.96%) were smokers with 3.4% alcohol users, 19.8% obese, and 8.9% with MS (Table 1). In the total cohort, 0.78% of hospitalizations (*n* = 18,926) had a diagnosis of HMB which included 15,180 (0.63%) hospitalizations without IM and 3746 (0.15%) with IM. Age was found to be a strong modifier for most CVD outcomes including diabetes mellitus (DM) (Additional file 1: Table S1) and thus results are presented for different age groups, separately.

**Table 1:** Sample characteristics of HMB among hospitalized women of age 18-70 years, NIS-2017

**Characteristics**	**Total**	**NMC**	**HMB**	***p*-value**
** Total**	**N=** **2430851**	**N=2411925 (99.22%)**	**N=18926 (0.78%)**	
** Age (years), **Mean (SD)	44.36 (15.97)	44.39 (16.01)	40.94 (7.99)	<0.001
** Race/Ethnicity**				<0.001
White	1409176 (57.97%)	1402255 (58.14%)	6921 (36.57%)	
Black	422067 (17.36%)	415435 (17.22%)	6632 (35.04%)	
Hispanic	326760 (13.44%)	323487 (13.41%)	3273 (17.29%)	
Others/Missing	272848 (11.22%)	270748 (11.23%)	2100 (11.10%)	
** HH income quartile**				<0.001
1st	759830 (31.26%)	753411 (31.24%)	6419 (33.92%)	
2nd	633632 (26.07%)	628750 (26.07%)	4882 (25.80%)	
3rd	554421 (22.81%)	550224 (22.81%)	4197 (22.18%)	
4th	447715 (18.42%)	444579 (18.43%)	3136 (16.57%)	
Missing	35253 (1.45%)	34961 (1.45%)	292 (1.54%)	
** Primary payer**				<0.001
Medicare	572996 (23.57%)	572008 (23.72%)	988 (5.22%)	
Medicaid	719175 (29.59%)	713922 (29.60%)	5253 (27.76%)	
Private insurance	957547 (39.39%)	947137 (39.27%)	10410 (55.00%)	
Self-Pay	103045 (4.24%)	101518 (4.21%)	1527 (8.07%)	
No charge	8163 (0.34%)	8010 (0.33%)	153 (0.81%)	
Other	69925 (2.88%)	69330 (2.87%)	595 (3.14%)	
** Smoking use**	23273 (0.96%)	23093 (0.96%)	180 (0.95%)	0.93
** Alcohol use**	83295 (3.43%)	82927 (3.44%)	368 (1.94%)	<0.001
** Obesity**	481599 (19.81%)	477229 (19.79%)	4370 (23.09%)	<0.001
** Metabolic syndrome **	216109 (8.89%)	215180 (8.92%)	929 (4.91%)	<0.001
** Contraceptive/Hormone use**	8136 (0.33%)	7942 (0.33%)	194 (1.03%)	<0.001
** Leiomyoma uterus**	39970 (1.64%)	30227 (1.25%)	9743 (51.48%)	<0.001
** Polycystic ovary syndrome**	13486 (0.55%)	13176 (0.55%)	310 (1.64%)	<0.001
** Anemia**	208235 (8.57%)	205269 (8.51%)	2966 (15.67%)	<0.001
** Anticoagulant use**	86299 (3.55%)	85693 (3.55%)	606 (3.20%)	0.010
** NSAIDs**	17303 (0.71%)	17096 (0.71%)	207 (1.09%)	<0.001
** Inflammatory bowel disease**	26691 (1.10%)	26533 (1.10%)	158 (0.83%)	<0.001
** Fasting Glucose**	425977 (17.52)	424070 (17.58%)	1907 (10.08%)	<0.001
** Infertility**	948 (0.04%)	849 (0.04%)	99 (0.52%)	<0.001
** Hypertension**	613784 (25.25%)	609300 (25.26%)	4484 (23.69%)	<0.001
** High Triglyceride**	402621 (16.56%)	401339 (16.64%)	1282 (6.77%)	<0.001
** Low high-density lipoprotein**	613 (0.03%)	593 (0.02%)	20 (0.11%)	<0.001
** Insulin use**	165117 (6.79%)	164500 (6.82%)	617 (3.26%)	<0.001

*Abbreviations*: *NIS* National inpatient sample, *SD* Standard deviation, *HH* Household, *NSAIDs* Non-steroidal anti-inflammatory drugs, *NMC* Normal menstrual cycle, *HMB* Heavy menstrual bleeding

All the baseline characteristics were found to be different between HMB and NMC groups except for smoking status. HMB was strongly associated with age, black race, and private insurance. The proportion of obesity (23.1% vs. 19.8%, *p* < 0.001), contraceptive use (1.03% vs. 0.33%, *p* < 0.001), PCOS (1.64% vs. 0.55%, *p* < 0.001), infertility (0.52% vs. 0.04%, *p* < 0.001), anemia (15.7% vs. 8.5%, *p* < 0.001), NSAIDs (1.09% vs 0.71% *p* < 0.001), and leiomyoma uterus (51.5% vs. 1.3%, *p* < 0.001) were significantly higher in HMB hospitalizations than in NMC group whereas MS, individual components of MS, insulin use, IBD, and DM were significantly lower in HMB compared to NMC (Table 1). However, HMB hospitalizations with age ≤ 40 years were associated with all the risk factors including DM, MS, insulin use, IBD, and infertility (Additional file 1: Table S2). The proportion of MACE was found highest (10.2%, *n* = 247,464) after DM (19.3%, *n* = 468,304) in our selected cohort, and the lowest proportion was observed for stroke (1.5%, *n* = 36,004) and myocardial infarction (1.6%, *n* = 39,899). All the CVD outcomes were significantly higher in proportions in the HMB group compared to the NMC group among hospitalizations of women with age ≤ 40 years (Additional file 1: Table S3).

### Unadjusted and propensity scores-matched associations of HMB diagnosis with CVD outcomes among hospitalizations of women with age ≤ 40 years

**Fig. 1:**
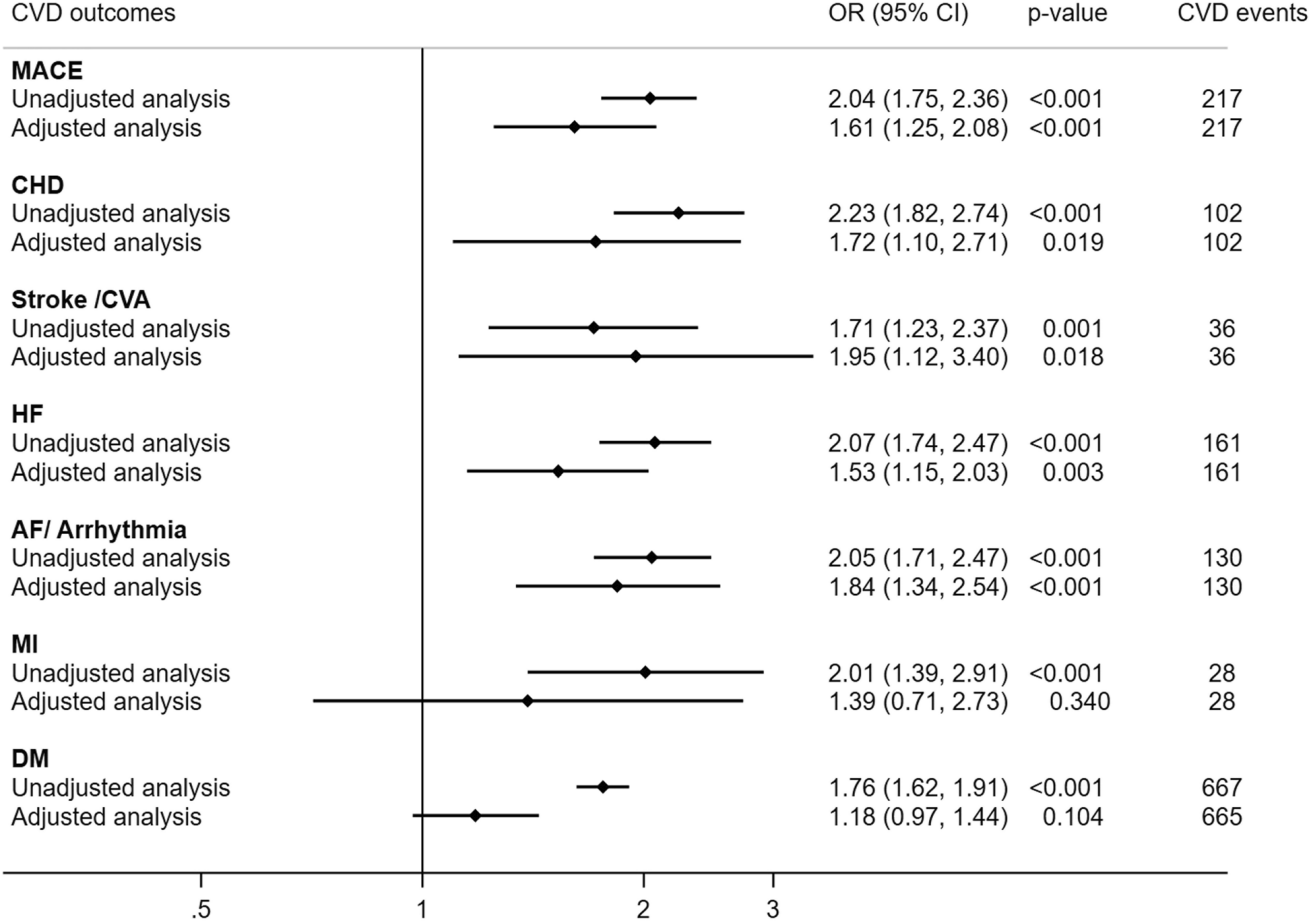
Unadjusted and adjusted associations of HMB with CVD outcomes including diabetes among hospitalized women of ages 18–40 years. OR, Odds ratio; CI, Confidence interval; CVD, Cardiovascular disease; MACE, Major adverse cardiovascular event; CHD, coronary heart disease; CVA, Cerebrovascular accident; HF, Heart failure; AF, Atrial fibrillation; MI, Myocardial infarction; DM, Diabetes mellitus; NMC, Normal menstrual cycle; HMB, Heavy menstrual bleeding. MACE was defined as the composite of myocardial infarction, stroke, and heart failure. The adjusted associations were carried out using propensity scores-matched analysis. The propensity scores model included race/ethnicity, household income quartile, primary payer, smoking, alcohol, contraceptive use, metabolic syndrome, non-steroidal anti-inflammatory drugs, leiomyoma uterus, obesity, polycystic ovary syndrome, anemia, and anticoagulants. Adjusted model additionally adjusted for age

In the unadjusted analyses, the odds of MACE, CHD, HF, AF or arrhythmia, and MI were twofold higher in HMB hospitalizations compared to hospitalizations without menstrual disorders. A 70% or higher odds of stroke/ CVA and DM were also observed in HMB hospitalizations compared to hospitalizations with NMC (Fig. 1). In propensity scores-matched analysis, HMB was significantly associated with increased odds of CVD outcomes including MACE (OR = 1.61; 95% CI: 1.25, 2.08, *p* < 0.001), CHD (OR = 1.72; 95% CI: 1.10, 2.71, p = 0.019), stroke/CVA (OR = 1.95; 95% CI: 1.12, 3.40, *p* = 0.018), HF (OR = 1.53; 95% CI: 1.15, 2.03, *p* = 0.003), and AF/arrhythmia (OR = 1.84; 95% CI: 1.34, 2.54, *p* < 0.001) (Fig. 1).

### Sensitivity analyses-adjusted association of HMB diagnosis with CVD outcomes among hospitalizations of women with age ≤ 40 years

After adjusting for age, race/ethnicity, household income quartile, primary payer, smoking, alcohol, contraceptive use, MS, NSAIDs, and leiomyoma uterus, all the outcomes including MACE, CHD, HF, AF, and DM were found to be significantly associated with HMB except for stroke/CVA and MI. The relative risk models also yielded the same pattern of effect sizes in the adjusted analyses (Additional file 1: Table S4). The adjusted association of HMB with each CVD outcome remained unchanged after additionally adjusting for fasting glucose, hypertension, high triglycerides, low HDL, obesity, pre-diabetes, DM, parity, anemia, and IBD (Additional file 1: Table S4). The associations obtained in primary propensity scores-matched analyses were unchanged after excluding hospitalization records with PCOS, leiomyoma uterus, or congenital heart disease (Additional file 1: Table S5) or removing anticoagulation use, NSAID use, or lifestyle cofactors (Additional file 1: Table S6) from the analyses. The associations were more pronounced after removing smoking/alcohol use and obesity (Additional file 1: Table S6).

### Adjusted direct and indirect associations of HMB diagnosis with CVD outcome through mediators among hospitalizations of women aged ≤ 40 years

In the mediation analysis, the HMB was directly associated with MACE after accounting for mediating association of MS (OR = 1.45; 95%CI: 1.27, 1.66), obesity (OR = 1.41; 95%CI: 1.18, 1.67), hypertension (OR = 1.37; 95%CI: 1.17, 1.62), DM (OR = 1.46; 95%CI: 1.23, 1.74), and anemia (OR = 1.47; 95%CI: 1.24, 1.74). The indirect association of HMB with MACE was less pronounced compared to the direct association of HMB with MACE after adjusting for age, race/ethnicity, household income quartile, primary payer, smoking, alcohol, contraceptive use, MS, NSAIDs, and leiomyoma uterus (Additional file 1: Table S7).

### Associations of all covariates with CVD outcome among hospitalizations of women aged ≤ 40 years

None of the lifestyle covariates had a significant interaction with HMB on most CVD outcomes (Additional file 1: Table S8). Most of the considered covariates were found to be associated with MACE except for infertility, IBD, and NSAIDs. In the adjusted analysis, anticoagulant use (OR = 5.31; 95%CI: 4.91, 5.74), black race/ethnicity (OR = 2.05; 95%CI: 1.94, 2.16), insulin use (OR = 2.53; 95%CI: 2.37, 2.71), contraceptive/hormone use (OR = 1.85; 95%CI: 1.46, 2.35), obesity (OR = 1.84; 95%CI: 1.76, 1.93), MS(OR = 1.80; 95%CI: 1.66, 1.96), smoking use (OR = 1.65; 95%CI: 1.42, 1.91), anemia (OR = 1.30; 95%CI: 1.22, 1.37), and alcohol use (OR = 1.11; 95%CI: 1.00, 1.23) were associated with increased odds of MACE events in addition to HMB (OR = 1.30; 95%CI: 1.10, 1.54) (Additional file 1: Table S9).

### Unadjusted and adjusted associations of HMB with or without IM with CVD outcomes among hospitalizations of women with age ≤ 40 years

In the unadjusted analyses, HMB with or without IM was associated with all CVD outcomes except for MI. In the propensity scores-matched analyses, HMB without IM was associated with MACE (OR = 1.69; 95%CI: 1.30, 2.19, *p* < 0.001), CHD (OR = 1.67; 95%CI: 1.05, 2.66, *p* = 0.031), stroke/CVA (OR = 1.96; 95%CI: 1.11, 3.47, *p* = 0.021), HF (OR = 1.60; 95%CI: 1.11, 3.47, *p* = 0.002), and AF (OR = 1.84; 95%CI: 1.31, 2.57, *p* < 0.001). However, HMB with IM was only associated with CHD (OR = 1.95; 95%CI: 1.10, 3.48, *p* = 0.023) and AF (OR = 1.87; 95%CI: 1.17, 2.9, *p* = 0.009) (Table 2). These associations other than stroke/CVA remained consistent in adjusted regression models or after additionally adjusting for DM (Additional file 1: Table S10).

**Table 2:** Unadjusted adjusted associations of HMB with or without IM and CVD events including diabetes among hospitalized women of ages between 18-40 years

	**Unadjusted analysis (N=1139431)**		***p*-value**	PS-Model* (N=15440)		***p*-value**
	**CVD events (%)**	**OR (95% CI)**		**CVD events (%)**	**OR (95% CI)**	
** MACE**						
NMC (reference)	15764 (1.39%)	1		121 (1.57%)	1	
Only HMB	182 (2.92%)	2.13 (1.81, 2.50)	<0.001	182 (2.93%)	1.69 (1.30, 2.19)	<0.001
HMB with IM	35 (2.30%)	1.67 (1.19, 2.34)	0.003	35 (2.31%)	1.30 (0.87, 1.95)	0.198
** CHD**						
NMC (reference)	6706 (0.59%)	1		48 (0.62%)	1	
Only HMB	79 (1.27%)	2.15 (1.71, 2.71)	<0.001	79 (1.27%)	1.67 (1.05, 2.66)	0.031
HMB with IM	23 (1.51%)	2.57 (1.71, 3.86)	<0.001	23 (1.52%)	1.95 (1.10, 3.48)	0.023
** Stroke /CVA**						
NMC (reference)	3080 (0.27%)	1		18 (0.23%)	1	
Only HMB	29 (0.46%)	1.71 (1.19, 2.46)	0.004	29 (0.47%)	1.96 (1.11, 3.47)	0.021
HMB with IM	7 (0.46%)	1.69 (0.80, 3.56)	0.167	7 (0.46%)	1.93 (0.80, 4.67)	0.146
** HF**						
NMC (reference)	11436 (1.01%)	1		93 (1.20%)	1	
Only HMB	135 (2.16%)	2.17 (1.79, 2.62)	<0.001	135 (2.18%)	1.60 (1.20, 2.15)	0.002
HMB with IM	26 (1.71%)	1.70 (1.15, 2.52)	0.008	26 (1.71%)	1.24 (0.79, 1.95)	0.354
** AF/ Arrhythmia**						
NMC (reference)	9309 (0.82%)	1		68 (0.88%)	1	
Only HMB	104 (1.67%)	2.04 (1.65, 2.53)	<0.001	104 (1.68%)	1.84 (1.31, 2.57)	<0.001
HMB with IM	26 (1.71%)	2.09 (1.42, 3.08)	<0.001	26 (1.71%)	1.87 (1.17, 2.99)	0.009
** MI**						
NMC (reference)	2031 (0.18%)	1		16 (0.21%)	1	
Only HMB	24 (0.38%)	2.15 (1.44, 3.2)	<0.001	24 (0.39%)	1.49 (0.74, 2.97)	0.261
HMB with IM	4 (0.26%)	1.46 (0.55, 3.9)	0.445	4 (0.26%)	0.99 (0.32, 3.07)	0.988
** DM**						
NMC (reference)	57369 (5.07%)	1		534 (6.92%)	1	
Only HMB	550 (8.82%)	1.81 (1.65, 1.99)	<0.001	548 (8.84%)	1.22 (0.99, 1.49)	0.061
HMB with IM	117 (7.68%)	1.56 (1.29, 1.88)	<0.001	117 (7.71%)	1.04 (0.80, 1.35)	0.773

*Abbreviations*: *OR *Odds ratio, *CI *Confidence interval, *CVD *Cardiovascular disease, *MACE *Major adverse cardiovascular event, *CHD *coronary heart disease, *CVA *Cerebrovascular accident, *HF *Heart failure, *AF *Atrial fibrillation, *MI *Myocardial infarction, *DM *Diabetes mellitus, *PCOS *Polycystic ovary syndrome, *NMC *Normal menstrual cycle, *HMB *Heavy menstrual bleeding, *IM *Irregular mensuration, *PS *Propensity scores, *NSAIDs *Non-steroidal anti-inflammatory drugs. MACE was defined as the composite of myocardial infarction, stroke, and heart failure

*The adjusted associations were carried out using propensity scores-matched analyses (PS-model). Propensity scores model included race/ethnicity, household income quartile, primary payer, smoking, alcohol, contraceptive use, metabolic syndrome, NSAIDs, leiomyoma uterus, obesity, PCOS, anemia, and anticoagulants; Model additionally adjusted for age on the PS-matched samples

### Unadjusted and adjusted associations of HMB diagnosis with CVD outcomes among hospitalizations of women with age > 40 years

In the unadjusted analyses, HMB was inversely associated with all CVD outcomes. However, HMB was only found positively associated with MACE (OR = 1.19; 95% CI: 1.01, 1.42, *p* = 0.032), HF (OR = 1.19; 95% CI: 1.01, 1.40, *p* = 0.04), and MI (OR = 1.46; 95% CI: 1.04, 2.04, *p* = 0.027) in the propensity scores-matched analysis (Additional file 1: Table S11). These findings remained significant among hospitalizations of women aged > 55 years but not in those aged between 40 to 55 years. HMB was associated with AF/ Arrhythmia (OR = 1.96; 95%CI: 1.24, 3.09) only in ages between 40 to 55 years while HMB was also significantly associated with DM (OR = 1.12; 95%CI: 1.01, 1.24) among hospitalizations of women aged > 55 years (Additional file 1: Table S12).

## Discussion

We observed a notable increase in MACE, CHD, HF, AF/arrhythmia, stroke/CVA, and DM associated with HMB among hospitalizations of young women. Most of the CVD outcomes remained significantly associated with HMB even after additionally accounting for individual components of MS, insulin use, DM, IBD, infertility, and anemia among hospitalizations of young women. The associations were more pronounced with HMB alone compared to HMB with IM in hospitalizations of women with age ≤ 40 years. In addition, we also observed some CVD events including MACE, HF, and MI associated with HMB among hospitalizations of women with age > 40 years.

A few studies recognized that HMB is associated with IM, hirsutism, PCOS, and secondary amenorrhea among teenagers and young women, which may lead to insulin resistance and other metabolic abnormalities and subsequently CVD [16, 17, 26]. However, in our study, HMB alone was strongly associated with most CVD outcomes compared to HMB with IM. We also observed an association between HMB and CVD outcomes after excluding PCOS cases. These findings suggest that HMB without comorbid IM or PCOS itself is an important factor for CVD outcomes among young women. It has been shown that HMB could be linked to the use of anticoagulation and NSAIDs [9]. However, our analysis infers that HMB is associated with CVD outcomes regardless of anticoagulation or NSAID use among young women. A higher prevalence of HMB has been reported in women with congenital heart disease. [27] In our study, the association remained consistent between HMB and CVD outcomes even after excluding congenital heart disease. Several studies reported that obesity, metabolic disorders, and anemia are strongly associated with HMB, which may increase the risk of CVD and other associated adverse health outcomes [11, 15, 18, 28]. However, we noticed that the association of HMB with CVD outcomes among young women was independent of obesity, MS, anemia, insulin use, and DM. Although MS or visceral obesity index, hypertension, insulin use, DM, and anemia are considered the potential mediators for the association of HMB and CVD outcomes[4, 16, 29], the direct association of HMB with CVD outcome was markedly higher than the indirect association of HMB with CVD through these mediators in our mediation analyses. These findings directly confirm a potential association between CVD events and HMB condition, particularly among women with age ≤ 40 years indicating that HMB is an early sign and critical factor for CVD outcomes. However, a longitudinal study is required to confirm the age at the onset of the HMB effect on the risk of CVD development.

Contrary to findings observed in young women, HMB was not robustly associated with CVD events in the analysis of hospitalized women of age > 40 years in the primary analysis. This could be due to the predominantly presence of uterine fibroids among peri-and postmenopausal women compared to women with age ≤ 40 years. HMB has been shown to be associated with uterine fibroids [30]. In the analysis of women with age ≤ 40 years, the association between HMB and CVD outcomes was unchanged even after excluding the leiomyoma uterus. However, HMB was inversely associated with CVD outcomes without adjusting for leiomyoma uterus in the analysis of records with age > 40 years. In this age group, the average age of HMB was 46 years suggesting that CVD risk associated with HMB is due to uterine fibroids among perimenopausal women. In our sensitivity analysis after removing hospitalization records of peri- or menopausal transition women, HMB remained associated with MACE due to its association with HF only indicating a history of HMB may be a critical factor for HF in postmenopausal women. One study found that women diagnosed with menorrhagia and who underwent hysterectomy had increased levels of serum inflammatory markers, predisposing them to future cardiovascular events [29]. In another cohort study [31], a higher risk of CVD and adverse metabolic health was observed among women who underwent a hysterectomy with ovarian conservation compared to those who did not. Although our findings were consistent after accounting for the exogenous use of contraceptives/hormones, HMB-associated hormonal changes and inflammation may be the primary reasons for CVD events, particularly among young women.

Similar to our study, advancing reproductive age, black race, lower household income, and obesity were found to be associated with HMB in previous studies [11]. In addition to HMB, insulin use, MS, hormonal use, and anemia were also strongly associated with CVD in our analysis of hospitalizations of young women indicating that iron deficiency, insulin resistance, and hormonal changes along with HMB are important factors of CVD risk. Recent studies also highlighted the impact of coronavirus disease-19 on HMB condition due to stress-induced effects on hypothalamic function and the presence of the virus receptor and angiotensin-converting enzyme in the woman’s uterus and ovary [32]. This suggests a link between inflammation, mental health, and CVD outcomes among women with menstrual disorders, especially HMB. Although the precise mechanisms for HMB-associated cardiac abnormalities are unknown, the main reasons could be the hormonal dysregulation related to progesterone, estradiol, glucocorticoids, and androgens leading to hypoxia, inflammation, impaired vasoconstriction, and altered hemostasis [11]. In HMB patients, reduced expression of hypoxia-inducible factor (HIF)α, the proliferation of vascular smooth muscle, and transforming growth factor β1 are linked with hypoxia and menstrual repair [33]. Increased expression of 11β-hydroxysteroid dehydrogenase in HMB patients seems to affect cortisol and vasoconstriction [34]. In addition, reduced expression of HOXA10 and 11 genes, altered function of estrogen receptor β and progesterone receptor, and increased matrix metalloproteinases and nuclear factor kappa B activities have been reported to influence inflammation [35, 36]. In HMB patients, increased levels of tissue plasminogen activator affecting hemostasis, fibrin, and D-dimers have also been reported [11, 37]. The related treatments such as hormonal-based treatments including combined oral contraceptives or progestin-only contraceptives, and hemostatic therapy affecting these pathways have been developed for HMB [8, 9, 15]. Although the use of these treatments for HMB including hysterectomy depends on multiple factors including age, HMB pathology, fertility status, and desire to become pregnant[9], effective treatment strategies are needed to be developed for HMB management considering side effects of hormonal therapy [8, 11, 38]. In addition, a reduction of environmental exposure is also useful for minimizing the risk of menstrual disorders and CVD events [3, 39]. These suggest that HMB may be associated with CVD outcomes through environmental stressors, stress-induced inflammation, hormonal changes, oxidative stress, and hemodynamic instability.

One of the major limitations of our study is the cross-sectional study design using hospitalization records, which limits the generalizability of our study findings. Our study does not allow us to infer the causality of the association between HMB and CVD outcomes. Owing to a retrospective analysis of a secondary database, we were unable to account for some confounders including the type of anticoagulants and tranexamic acid use, dietary pattern, family history, or other comorbidities, and the onset of menorrhagia. Although HMB might likely have occurred before CVD events as this study is based on hospitalization records, the lack of a time gap between HMB and CVD events prohibited us from estimating the CVD risk associated with HMB. The lack of information on HMB and IM across the reproductive lifespan further limits the interpretation of our findings. Moreover, the ICD-10 codes may not fully capture MS and may underestimate HMB diagnosis among all hospitalizations leading to biased associations. The coding biases and data integrity issues in the NIS database may also negatively influence the findings obtained in our study. Despite these limitations, our study attempted to address a critical question for improving women’s CVD health utilizing a large population database representing US hospitalizations. Moreover, this large NIS database allowed us to evaluate the relatively lower frequency of CVD outcomes among young women. The comprehensive inclusion of CVD events and rigorous analyses including propensity-matched and survey-weighted logistic regression adjusted analyses of HMB with or without IM with comprehensive adjustment of covariates provide valuable contributions to the limited literature on HMB and CVD health. Our multiple sensitivity and mediation analyses further validate the robustness of the findings reported in our study. Furthermore, the moderate to large effect sizes for the association between HMB and CVD events obtained in our study signify the role of HMB-associated CVD events among young women.

## Conclusions

In our study, HMB was strongly associated with CVD outcomes regardless of obesity, metabolic disorders, hormonal use, anemia, and uterine fibroids among US hospitalizations of young women. In addition, HMB without IM was profoundly associated with most CVD outcomes among US hospitalizations of young women. However, HMB was not robustly associated with CVD events among hospitalized women with age > 40 years. Future cohort studies are required to evaluate the age at onset of HMB and its long-term effect on the development of CVD outcomes. Our findings suggest promoting increased awareness, early detection, and optimum management of HMB to avoid adverse consequences of HMB including CVD outcomes among young females.

### Supplementary Information

Additional file 1: Table S1. Age as a modifier for the association between HMB and each CVD outcome including diabetes among hospitalized women of ages between 18-70 years. Table S2. Sample characteristics of HMB among hospitalized women by age groups. Table S3. Prevalence of CVD events including diabetes among hospitalized women by age groups. Table S4. Adjusted association of HMB with each CVD outcome including diabetes among hospitalized women of ages between 18-40 years using survey-weighted logistic and Poisson regression analyses. Table S5. Adjusted association of HMB with CVD events including diabetes after excluding PCOS, leiomyoma uterus, and congenital heart disease cases among hospitalized women of ages between 18-40 years using survey-weighted logistic regression analyses. Table S6. Adjusted association of HMB with CVD events including diabetes after excluding anticoagulant use, NSAID use, and lifestyle factors among hospitalized women of ages between 18-40 years using survey-weighted logistic regression analyses. Table S7. Adjusted direct and indirect associations of HMB diagnosis with CVD outcome through mediators among hospitalizations of women aged ≤40 years using survey-weighted logistic regression analyses. Table S8. Interaction between lifestyle factors and HMB on CVD outcomes among hospitalized women of ages between 18-40 years using survey-weighted logistic regression analyses. Table S9. Adjusted factors associated with MACE outcome among hospitalized women of ages between 18-40 years using survey-weighted logistic regression analyses. Table S10. Adjusted association between HMB categories with CVD events including diabetes among hospitalized women of ages 18-40 years using survey-weighted logistic regression analyses. Table S11. Unadjusted and adjusted associations of HMB with CVD outcomes including diabetes among hospitalized women of age >40 years. Table S12. Adjusted associations of HMB with CVD outcomes including diabetes among hospitalized women according to age group using propensity scores-matched analyses

## Data Availability

The dataset used in this study can be purchased from the HCUP-Agency for Healthcare Research and Quality (AHRQ) website (https://hcup-us.ahrq.gov/nisoverview.jsp). The study spreadsheets generated from NIS and related statistical codes may be obtained from the corresponding author upon appropriate request.

## Abbreviations

AF: Atrial fibrillation
CHD: Coronary heart disease
CI: Confidence interval
CVA: Cardiovascular accident
CVD: Cardiovascular disease
DM: Diabetes mellitus
HCUP: Healthcare Cost and Utilization Project
HF: Heart failure
HH: Household
HMB: Heavy menstrual bleeding
IBD: Inflammatory bowel disease
ICD-10: International Classification of Diseases, 10th Revision
IM: Irregular menstruation
MACE: Major adverse cardiovascular events
MI: Myocardial infarction
MS: Metabolic syndrome
NMC: Normal menstrual cycle
NSAIDs: Non-steroidal anti-inflammatory drugs
OR: Odds ratio
PCOS: Polycystic ovary syndrome
RR: Relative risk
