# Supplementary material for: Association of heavy menstrual bleeding with cardiovascular disease in US female hospitalizations
Source: BMC Med. 2024 May 23;22:208. doi: 10.1186/s12916-024-03426-8 (PMC11119710; doi:10.1186/s12916-024-03426-8)
Supplement: Supplementary file 1 — Additional file 1: Table S1. Age as a modifier for the association between HMB and each CVD outcome including diabetes among hospitalized women of ages between 18-70 years. Table S2. Sample characteristics of HMB among hospitalized women by age groups. Table S3. Prevalence of CVD events including diabetes among hospitalized women by age groups. Table S4. Adjusted association of HMB with each CVD outcome including diabetes among hospitalized women of ages between 18-40 years using survey-weighted logistic and Poisson regression analyses. Table S5. Adjusted association of HMB with CVD events including diabetes after excluding PCOS, leiomyoma uterus, and congenital heart disease cases among hospitalized women of ages between 18-40 years using survey-weighted logistic regression analyses. Table S6. Adjusted association of HMB with CVD events including diabetes after excluding anticoagulant use, NSAID use, and lifestyle factors among hospitalized women of ages between 18-40 years using survey-weighted logistic regression analyses. Table S7. Adjusted direct and indirect associations of HMB diagnosis with CVD outcome through mediators among hospitalizations of women aged ≤40 years using survey-weighted logistic regression analyses. Table S8. Interaction between lifestyle factors and HMB on CVD outcomes among hospitalized women of ages between 18-40 years using survey-weighted logistic regression analyses. Table S9. Adjusted factors associated with MACE outcome among hospitalized women of ages between 18-40 years using survey-weighted logistic regression analyses. Table S10. Adjusted association between HMB categories with CVD events including diabetes among hospitalized women of ages 18-40 years using survey-weighted logistic regression analyses. Table S11. Unadjusted and adjusted associations of HMB with CVD outcomes including diabetes among hospitalized women of age >40 years. Table S12. Adjusted associations of HMB with CVD outcomes including diabetes among hospitalized [file 12916_2024_3426_MOESM1_ESM.docx]

# **Supplementary Tables**

**Association of heavy menstrual bleeding with cardiovascular disease in US female hospitalizations**

Pallavi Dubey, PhD^1^ Sireesha Reddy, MD^1^ Vishwajeet Singh, PhD^2^ Abdelrehman Yousif, MD^1^ and Alok Kumar Dwivedi, PhD^2,3*^

^1^Department of Obstetrics and Gynecology, Paul L. Foster School of Medicine, Texas Tech University Health Sciences Center El Paso, El Paso, Texas, US

^2^Biostatistics and Epidemiology Consulting Lab, Office of Research, Texas Tech University Health Sciences Center El Paso, El Paso, Texas, USA

^3^Division of Biostatistics & Epidemiology, Department of Molecular and Translational Medicine, Paul L. Foster School of Medicine, Texas Tech University Health Sciences Center El Paso, El Paso, Texas, USA

**Supplementary Table 1. Age as a modifier for the association between HMB and CVD outcomes including diabetes among hospitalized women of ages between 18-70 years**

**Supplementary Table 2. Sample characteristics of HMB among hospitalized women by age groups**

**Supplementary Table 3. Prevalence of CVD events including diabetes among hospitalized women by age groups**

**Supplementary Table 4. Adjusted association of HMB with each CVD outcome including diabetes among hospitalized women of ages between 18-40 years using survey-weighted logistic and Poisson regression analyses**

**Supplementary Table 5. Adjusted association of HMB with CVD events including diabetes after excluding PCOS, leiomyoma uterus, and congenital heart disease cases among hospitalized women of ages between 18-40 years using survey-weighted logistic regression analyses**

**Supplementary Table 6. Adjusted association of HMB with CVD events including diabetes after excluding anticoagulant use, NSAID use, and lifestyle factors among hospitalized women of ages between 18-40 years using survey-weighted logistic regression analyses**

**Supplementary Table 7. Adjusted direct and indirect associations of HMB diagnosis with CVD outcome through mediators among hospitalizations of women aged ≤40 years using survey-weighted logistic regression analyses**

**Supplementary Table 8. Interaction between lifestyle factors and HMB on CVD outcomes among hospitalized women of ages 18-40 years using survey-weighted logistic regression analyses**

**Supplementary Table 9. Adjusted factors associated with MACE outcome among hospitalized women of ages between 18-40 years using survey-weighted logistic regression analyses**

**Supplementary Table 10. Adjusted association between HMB categories with CVD events including diabetes among hospitalized women of ages 18-40 years using survey-weighted logistic regression analyses**

**Supplementary Table 11. Unadjusted and adjusted associations of HMB with CVD outcomes including diabetes among hospitalized women of age >40 years**

**Supplementary Table 12. Adjusted associations of HMB with CVD outcomes including diabetes among hospitalized women according to age group using propensity scores-matched analyses**

**Supplementary Table 1. Age as a modifier for the association between HMB and each CVD outcome including diabetes among hospitalized women of ages between 18-70 years**

|  | **Main effect** | | | **Interaction with age** | | |
| --- | --- | --- | --- | --- | --- | --- |
|  | **OR (95% CI)** | **p-value** | **OR (95% CI)** | | **p-value** |  |
| **MACE** |  |  |  | |  |  |
| NMC (reference) | 1 |  |  | |  |  |
| HMB | 2.01 (1.21, 3.33) | 0.007 | 0.98 (0.97, 0.99) | | <0.001 |  |
| **CHD** |  |  |  | |  |  |
| NMC (reference) | 1 |  |  | |  |  |
| HMB | 0.89 (0.48, 1.64) | 0.702 | 0.99 (0.98, 1.01) | | 0.251 |  |
| **Stroke /CVA** |  |  |  | |  |  |
| NMC (reference) | 1 |  |  | |  |  |
| HMB | 2.44 (0.69, 8.61) | 0.166 | 0.97 (0.94, 1.00) | | 0.046 |  |
| **HF** |  |  |  | |  |  |
| NMC (reference) | 1 |  |  | |  |  |
| HMB | 2.11 (1.17, 3.81) | 0.013 | 0.97 (0.96, 0.99) | | <0.001 |  |
| **AF/ Arrhythmia** |  |  |  | |  |  |
| NMC (reference) | 1 |  |  | |  |  |
| HMB | 6.82 (3.08, 15.13) | <0.001 | 0.95 (0.93, 0.97) | | <0.001 |  |
| **MI** |  |  |  | |  |  |
| NMC (reference) | 1 |  |  | |  |  |
| HMB | 0.40 (0.14, 1.19) | 0.099 | 1.01 (0.99, 1.04) | | 0.226 |  |
| **DM** |  |  |  | |  |  |
| NMC (reference) | 1 |  |  | |  |  |
| HMB | 2.47 (1.86, 3.29) | <0.001 | 0.97 (0.97, 0.98) | | <0.001 |  |

Abbreviations: OR, Odds ratio; CI, Confidence interval; CVD, Cardiovascular disease; MACE, Major adverse cardiovascular event; CHD, coronary heart disease; CVA, Cerebrovascular accident; HF, Heart failure; AF, Atrial fibrillation; MI, Myocardial infarction; DM, Diabetes mellitus; NMC, Normal menstrual cycle; HMB, Heavy menstrual bleeding. MACE was defined as the composite of myocardial infarction, stroke, and heart failure.

**Supplementary Table 2. Sample characteristics of HMB among hospitalized women by age groups**

| **Characteristics** | **Age ≤40 years** | | | | **Age >40 years** | | | |
| --- | --- | --- | --- | --- | --- | --- | --- | --- |
|  | **NMC** | **HMB** | **p-value** | **NMC** | | **HMB** | **p-value** |  |
|  | **N=1131669**  **(99.32%)** | **N=7762**  **(0.68%)** |  | **N=1280256**  **(99.14%)** | | **N=11164**  **(0.86%)** |  |  |
| **Age (years),** Mean (SD) | 29.20 (5.80) | 33.20 (5.79) | <0.001 | 57.81 (8.31) | | 46.32 (3.78) | <0.001 |  |
| **Race/Ethnicity** |  |  | <0.001 |  | |  | <0.001 |  |
| White | 576472 (50.94%) | 2693 (34.69%) |  | 825783 (64.50%) | | 4228 (37.87%) |  |  |
| Black | 194002 (17.14%) | 2915 (37.55%) |  | 221433 (17.30%) | | 3717 (33.29%) |  |  |
| Hispanic | 201393 (17.80%) | 1335 (17.20%) |  | 122094 (9.54%) | | 1938 (17.36%) |  |  |
| Others/Missing | 159802 (14.12%) | 819 (10.55%) |  | 110946 (8.67%) | | 1281 (11.47%) |  |  |
| **HH income quartile** |  |  | <0.001 |  | |  | 0.05 |  |
| 1st | 340834 (30.12%) | 2872 (37.00%) |  | 412577 (32.23%) | | 3547 (31.77%) |  |  |
| 2nd | 291911 (25.79%) | 2053 (26.45%) |  | 336839 (26.31%) | | 2829 (25.34%) |  |  |
| 3rd | 264006 (23.33%) | 1672 (21.54%) |  | 286218 (22.36%) | | 2525 (22.62%) |  |  |
| 4th | 221449 (19.57%) | 1063 (13.69%) |  | 223130 (17.43%) | | 2073 (18.57%) |  |  |
| Missing | 13469 (1.19%) | 102 (1.31%) |  | 21492 (1.68%) | | 190 (1.70%) |  |  |
| **Primary payer** |  |  | <0.001 |  | |  | <0.001 |  |
| Medicare | 41112 (3.63%) | 361 (4.65%) |  | 530896 (41.47%) | | 627 (5.62%) |  |  |
| Medicaid | 481103 (42.51%) | 2699 (34.77%) |  | 232819 (18.19%) | | 2554 (22.88%) |  |  |
| Private insurance | 519603 (45.91%) | 3703 (47.71%) |  | 427534 (33.39%) | | 6707 (60.08%) |  |  |
| Self-Pay | 51439 (4.55%) | 695 (8.95%) |  | 50079 (3.91%) | | 832 (7.45%) |  |  |
| No charge | 3095 (0.27%) | 68 (0.88%) |  | 4915 (0.38%) | | 85 (0.76%) |  |  |
| Other | 35317 (3.12%) | 236 (3.04%) |  | 34013 (2.66%) | | 359 (3.22%) |  |  |
| **Smoking status** |  |  | <0.001 |  | |  | <0.001 |  |
| No | 1125128 (99.42%) | 7674 (98.87%) |  | 1263704 (98.71%) | | 11072 (99.18%) |  |  |
| Yes | 6541 (0.58%) | 88 (1.13%) |  | 16552 (1.29%) | | 92 (0.82%) |  |  |
| **Alcohol** |  |  | 0.86 |  | |  | <0.001 |  |
| No | 1106252 (97.75%) | 7590 (97.78%) |  | 1222746 (95.51%) | | 10968 (98.24%) |  |  |
| Yes | 25417 (2.25%) | 172 (2.22%) |  | 57510 (4.49%) | | 196 (1.76%) |  |  |
| **Obesity** |  |  | <0.001 |  | |  | <0.001 |  |
| No | 982963 (86.86%) | 5950 (76.66%) |  | 951733 (74.34%) | | 8606 (77.09%) |  |  |
| Yes | 148706 (13.14%) | 1812 (23.34%) |  | 328523 (25.66%) | | 2558 (22.91%) |  |  |
| **MS** |  |  | <0.001 |  | |  | <0.001 |  |
| No | 1118904 (98.87%) | 7523 (96.92%) |  | 1077841 (84.19%) | | 10474 (93.82%) |  |  |
| Yes | 12765 (1.13%) | 239 (3.08%) |  | 202415 (15.81%) | | 690 (6.18%) |  |  |
| **Contraceptive/Hormone use** |  |  | <0.001 |  | |  | <0.001 |  |
| No | 1127503 (99.63%) | 7655 (98.62%) |  | 1276480 (99.71%) | | 11077 (99.22%) |  |  |
| Yes | 4166 (0.37%) | 107 (1.38%) |  | 3776 (0.29%) | | 87 (0.78%) |  |  |
| **PCOS** |  |  | <0.001 |  | |  | <0.001 |  |
| No | 1120784 (99.04%) | 7522 (96.91%) |  | 1277965 (99.82%) | | 11094 (99.37%) |  |  |
| Yes | 10885 (0.96%) | 240 (3.09%) |  | 2291 (0.18%) | | 70 (0.63%) |  |  |
| **Anemia** |  |  | <0.001 |  | |  | <0.001 |  |
| No | 1043716 (92.23%) | 6671 (85.94%) |  | 1162940 (90.84%) | | 9289 (83.20%) |  |  |
| Yes | 87953 (7.77%) | 1091 (14.06%) |  | 117316 (9.16%) | | 1875 (16.80%) |  |  |
| **Diabetes** |  |  | <0.001 |  | |  | <0.001 |  |
| No | 1074300 (94.93%) | 7095 (91.41%) |  | 871427 (68.07%) | | 9725 (87.11%) |  |  |
| Yes | 57369 (5.07%) | 667 (8.59%) |  | 408829 (31.93%) | | 1439 (12.89%) |  |  |
| **Anticoagulant** |  |  | <0.001 |  | |  | <0.001 |  |
| No | 1121290 (99.08%) | 7509 (96.74%) |  | 1204942 (94.12%) | | 10811 (96.84%) |  |  |
| Yes | 10379 (0.92%) | 253 (3.26%) |  | 75314 (5.88%) | | 353 (3.16%) |  |  |
| **NSAIDs** |  |  | <0.001 |  | |  | 0.90 |  |
| No | 1128729 (99.74%) | 7677 (98.90%) |  | 1266100 (98.89%) | | 11042 (98.91%) |  |  |
| Yes | 2940 (0.26%) | 85 (1.10%) |  | 14156 (1.11%) | | 122 (1.09%) |  |  |
| **Leiomyoma uterus** |  |  | <0.001 |  | |  | <0.001 |  |
| No | 1117728 (98.77%) | 5005 (64.48%) |  | 1263970 (98.73%) | | 4178 (37.42%) |  |  |
| Yes | 13941 (1.23%) | 2757 (35.52%) |  | 16286 (1.27%) | | 6986 (62.58%) |  |  |
| **Insulin use** |  |  | 0.0117 |  | |  | <0.001 |  |
| No | 1102624 (97.4%) | 7525 (96.9%) |  | 1144801 (89.4%) | | 10784 (96.6%) |  |  |
| Yes | 29045 (2.6%) | 237 (3.1%) |  | 135455 (10.6%) | | 380 (3.4%) |  |  |
| **IBD** |  |  | <0.001 |  | |  | <0.001 |  |
| No | 1125560 (99.5%) | 7693 (99.1%) |  | 1259832 (98.4%) | | 11075 (99.2%) |  |  |
| Yes | 6109 (0.5%) | 69 (0.9%) |  | 20424 (1.6%) | | 89 (0.8%) |  |  |
| **Infertility** |  |  | <0.001 |  | |  | <0.001 |  |
| No | 1130993 (99.9%) | 7699 (99.2%) |  | 1280083 (100.0%) | | 11128 (99.7%) |  |  |
| Yes | 676 (0.1%) | 63 (0.8%) |  | 173 (<1%) | | 36 (0.3%) |  |  |

Abbreviations: SD, Standard deviation; HH, Household; MS, Metabolic syndrome; PCOS: Polycystic ovary syndrome; NMC, Normal menstrual cycle; HMB, Heavy menstrual bleeding; NSAIDs, Non-steroidal anti-inflammatory drugs; IBD: inflammatory bowel disease.

**Supplementary Table 3. Prevalence of CVD events including diabetes among hospitalized women by age groups**

| **Outcomes** | **Total** | **Age ≤40 years** | | | **Age >40 years** | | |
| --- | --- | --- | --- | --- | --- | --- | --- |
|  |  | **NMC** | **HMB** | **p-value** | **NMC** | **HMB** | **p-value** |
|  | **N=2430851** | **N=1131669** | **N=7762** |  | **N=1280256** | **N=11164** |  |
| **MACE** |  |  |  | <0.001 |  |  | <0.001 |
| No | 2183387 (89.82%) | 1115905 (98.61%) | 7545 (97.20%) |  | 1049312 (81.96%) | 10625 (95.17%) |  |
| Yes | 247464 (10.18%) | 15764 (1.39%) | 217 (2.80%) |  | 230944 (18.04%) | 539 (4.83%) |  |
| **CHD** |  |  |  | <0.001 |  |  | <0.001 |
| No | 2220777 (91.36%) | 1124963 (99.41%) | 7660 (98.69%) |  | 1077368 (84.15%) | 10786 (96.61%) |  |
| Yes | 210074 (8.64%) | 6706 (0.59%) | 102 (1.31%) |  | 202888 (15.85%) | 378 (3.39%) |  |
| **Stroke/CVA** |  |  |  | 0.001 |  |  | <0.001 |
| No | 2394847 (98.52%) | 1128589 (99.73%) | 7726 (99.54%) |  | 1247446 (97.44%) | 11086 (99.30%) |  |
| Yes | 36004 (1.48%) | 3080 (0.27%) | 36 (0.46%) |  | 32810 (2.56%) | 78 (0.70%) |  |
| **HF** |  |  |  | <0.001 |  |  | <0.001 |
| No | 2239034 (92.11%) | 1120233 (98.99%) | 7601 (97.93%) |  | 1100426 (85.95%) | 10774 (96.51%) |  |
| Yes | 191817 (7.89%) | 11436 (1.01%) | 161 (2.07%) |  | 179830 (14.05%) | 390 (3.49%) |  |
| **AF/ Arrhythmia** |  |  |  | <0.001 |  |  | <0.001 |
| No | 2288944 (94.16%) | 1122360 (99.18%) | 7632 (98.33%) |  | 1148014 (89.67%) | 10938 (97.98%) |  |
| Yes | 141907 (5.84%) | 9309 (0.82%) | 130 (1.67%) |  | 132242 (10.33%) | 226 (2.02%) |  |
| **MI** |  |  |  | <0.001 |  |  | <0.001 |
| No | 2390952 (98.36%) | 1129638 (99.82%) | 7734 (99.64%) |  | 1242517 (97.05%) | 11063 (99.10%) |  |
| Yes | 39899 (1.64%) | 2031 (0.18%) | 28 (0.36%) |  | 37739 (2.95%) | 101 (0.90%) |  |
| **DM** |  |  |  | <0.001 |  |  | <0.001 |
| No | 1962547 (80.73%) | 1074300 (94.93%) | 7095 (91.41%) |  | 871427 (68.07%) | 9725 (87.11%) |  |
| Yes | 468304 (19.27%) | 57369 (5.07%) | 667 (8.59%) |  | 408829 (31.93%) | 1439 (12.89%) |  |

Abbreviations: CVD, Cardiovascular disease; MACE, Major adverse cardiovascular event; CHD, coronary heart disease; CVA, Cerebrovascular accident; HF, Heart failure; AF, Atrial fibrillation; MI, Myocardial infarction; DM, Diabetes mellitus; NMC, Normal menstrual cycle; HMB, Heavy menstrual bleeding. MACE was defined as the composite of myocardial infarction, stroke, and heart failure.

**Supplementary Table 4. Adjusted association of HMB with CVD events including diabetes among hospitalized women of ages between 18-40 years using survey-weighted logistic and Poisson regression analyses**

|  | **Adjusted model* (N=1139431)** | | **Adjusted model* (N=1139431)** | | **Adjusted model**(N=1139431)** | |
| --- | --- | --- | --- | --- | --- | --- |
|  | **OR (95% CI)** | **p-value** | **PR (95% CI)** | **p-value** | **OR (95% CI)** | **p-value** |
| **MACE** |  |  |  |  |  |  |
| NMC (reference) | 1 |  | 1 |  | 1 |  |
| HMB | 1.45 (1.23, 1.71) | <0.001 | 1.42 (1.22, 1.65) | <0.001 | 1.38 (1.17, 1.63) | <0.001 |
| **CHD** |  |  |  |  |  |  |
| NMC (reference) | 1 |  | 1 |  | 1 |  |
| HMB | 1.39 (1.11, 1.73) | 0.004 | 1.37 (1.11, 1.69) | 0.003 | 1.35 (1.07, 1.69) | 0.01 |
| **Stroke /CVA** |  |  |  |  |  |  |
| NMC (reference) | 1 |  | 1 |  | 1 |  |
| HMB | 1.33 (0.94, 1.88) | 0.109 | 1.32 (0.94, 1.86) | 0.108 | 1.12 (0.78, 1.59) | 0.542 |
| **HF** |  |  |  |  |  |  |
| NMC (reference) | 1 |  | 1 |  | 1 |  |
| HMB | 1.46 (1.21, 1.76) | <0.001 | 1.43 (1.2, 1.7) | <0.001 | 1.44 (1.18, 1.74) | <0.001 |
| **AF/ Arrhythmia** |  |  |  |  |  |  |
| NMC (reference) | 1 |  | 1 |  | 1 |  |
| HMB | 1.89 (1.55, 2.31) | <0.001 | 1.87 (1.54, 2.26) | <0.001 | 1.71 (1.41, 2.09) | <0.001 |
| **MI** |  |  |  |  |  |  |
| NMC (reference) | 1 |  | 1 |  | 1 |  |
| HMB | 1.34 (0.91, 1.97) | 0.136 | 1.33 (0.92, 1.94) | 0.134 | 1.26 (0.85, 1.85) | 0.247 |
| **DM** |  |  |  |  |  |  |
| NMC (reference) | 1 |  | 1 |  |  |  |
| HMB | 1.27 (1.15, 1.41) | <0.001 | 1.22 (1.14, 1.31) | <0.001 |  |  |

Abbreviations: OR, Odds ratio; PR, Prevalence ratio; CI, Confidence interval; CVD, Cardiovascular disease; MACE, Major adverse cardiovascular event; CHD, coronary heart disease; CVA, Cerebrovascular accident; HF, Heart failure; AF, Atrial fibrillation; MI, Myocardial infarction; DM, Diabetes mellitus; NMC, Normal menstrual cycle; HMB, Heavy menstrual bleeding. MACE was defined as the composite of myocardial infarction, stroke, and heart failure.

*Adjusted model included age, race/ethnicity, household income quartile, primary payer, smoking, alcohol, contraceptive use, metabolic syndrome, non-steroidal anti-inflammatory drugs, and leiomyoma uterus.

**Adjusted model included age, race/ethnicity, household income quartile, primary payer, smoking, alcohol, contraceptive use, non-steroidal anti-inflammatory drugs, leiomyoma uterus, hypertension, high triglyceride, fasting glucose, low high-density lipoprotein, insulin use, obesity, DM, inflammatory bowel disease, infertility, and anemia.

**Supplementary Table 5. Adjusted association of HMB with CVD events including diabetes after excluding PCOS, leiomyoma uterus, and congenital heart disease cases among hospitalized women of ages between 18-40 years using survey-weighted logistic regression analyses**

|  | **Excluding PCOS** | | **Excluding leiomyoma uterus** | | **Excluding congenital heart disease** | |
| --- | --- | --- | --- | --- | --- | --- |
|  | **OR (95% CI)*** | **p-value** | **OR (95% CI)**** | **p-value** | **OR (95% CI)***** | **p-value** |
| **MACE** |  |  |  |  |  |  |
| NMC (reference) | 1 |  | 1 |  | 1 |  |
| HMB | 1.48 (1.25, 1.75) | <0.001 | 1.51 (1.28, 1.79) | <0.001 | 1.45 (1.23, 1.71) | <0.001 |
| **CHD** |  |  |  |  |  |  |
| NMC (reference) | 1 |  | 1 |  | 1 |  |
| HMB | 1.43 (1.14, 1.78) | 0.002 | 1.30 (1.01, 1.67) | 0.044 | 1.39 (1.11, 1.73) | 0.004 |
| **Stroke /CVA** |  |  |  |  |  |  |
| NMC (reference) | 1 |  | 1 |  | 1 |  |
| HMB | 1.38 (0.97, 1.95) | 0.070 | 1.48 (1.05, 2.09) | 0.024 | 1.33 (0.94, 1.88) | 0.109 |
| **HF** |  |  |  |  |  |  |
| NMC (reference) | 1 |  | 1 |  | 1 |  |
| HMB | 1.48 (1.22, 1.79) | <0.001 | 1.48 (1.21, 1.81) | <0.001 | 1.46 (1.21, 1.77) | <0.001 |
| **AF/ Arrhythmia** |  |  |  |  |  |  |
| NMC (reference) | 1 |  | 1 |  | 1 |  |
| HMB | 1.88 (1.54, 2.3) | <0.001 | 2.14 (1.76, 2.6) | <0.001 | 1.88 (1.54, 2.29) | <0.001 |
| **MI** |  |  |  |  |  |  |
| NMC (reference) | 1 |  | 1 |  | 1 |  |
| HMB | 1.39 (0.94, 2.04) | 0.098 | 1.36 (0.91, 2.03) | 0.130 | 1.34 (0.91, 1.96) | 0.136 |
| **DM** |  |  |  |  |  |  |
| NMC (reference) | 1 |  | 1 |  | 1 |  |
| HMB | 1.27 (1.14, 1.41) | <0.001 | 1.32 (1.18, 1.48) | <0.001 | 1.27 (1.15, 1.41) | <0.001 |

Abbreviations: OR, Odds ratio; CI, Confidence interval; CVD, Cardiovascular disease; MACE, Major adverse cardiovascular event; CHD, coronary heart disease; CVA, Cerebrovascular accident; HF, Heart failure; AF, Atrial fibrillation; MI, Myocardial infarction; DM, Diabetes mellitus; PCOS: Polycystic ovary syndrome; NMC, Normal menstrual cycle; HMB, Heavy menstrual bleeding. MACE was defined as the composite of myocardial infarction, stroke, and heart failure.

* Adjusted model included age, race/ethnicity, household income quartile, primary payer, smoking, alcohol, contraceptive use, metabolic syndrome, non-steroidal anti-inflammatory drugs, and leiomyoma uterus after excluding PCOS cases.

** Adjusted model included age, race/ethnicity, household income quartile, primary payer, smoking, alcohol, contraceptive use, metabolic syndrome, non-steroidal anti-inflammatory drugs, and leiomyoma uterus after excluding leiomyoma uterus cases.

*** Adjusted model included age, race/ethnicity, household income quartile, primary payer, smoking, alcohol, contraceptive use, metabolic syndrome, non-steroidal anti-inflammatory drugs, and leiomyoma uterus after excluding congenital heart disease cases.

**Supplementary Table 6. Adjusted association of HMB with CVD events including diabetes after excluding anticoagulant and NSAID users among hospitalized women of ages between 18-40 years using survey-weighted logistic regression analyses**

|  | **Excluding anticoagulant** | | **Excluding NSAIDs** | | **Excluding Smoking/alcohol/obesity** | |
| --- | --- | --- | --- | --- | --- | --- |
|  | **OR (95% CI)*** | **p-value** | **OR (95% CI)**** | **p-value** | **OR (95% CI)***** | **p-value** |
| **MACE** |  |  |  |  |  |  |
| NMC (reference) | 1 |  | 1 |  | 1 |  |
| HMB | 1.37 (1.15, 1.64) | 0.001 | 1.47 (1.25, 1.74) | <0.001 | 1.57 (1.27, 1.95) | <0.001 |
| **CHD** |  |  |  |  |  |  |
| NMC (reference) | 1 |  | 1 |  | 1 |  |
| HMB | 1.31 (1.03, 1.66) | 0.028 | 1.39 (1.11, 1.74) | 0.004 | 1.60 (1.20, 2.12) | 0.001 |
| **Stroke /CVA** |  |  |  |  |  |  |
| NMC (reference) | 1 |  | 1 |  | 1 |  |
| HMB | 1.31 (0.91, 1.87) | 0.148 | 1.31 (0.92, 1.86) | 0.133 | 1.27 (0.82, 1.98) | 0.281 |
| **HF** |  |  |  |  |  |  |
| NMC (reference) | 1 |  | 1 |  | 1 |  |
| HMB | 1.36 (1.11, 1.68) | 0.003 | 1.48 (1.23, 1.80) | <0.001 | 1.59 (1.23, 2.05) | <0.001 |
| **AF/ Arrhythmia** |  |  |  |  |  |  |
| NMC (reference) | 1 |  | 1 |  | 1 |  |
| HMB | 1.53 (1.24, 1.90) | <0.001 | 1.92 (1.58, 2.34) | <0.001 | 2.22 (1.76, 2.80) | <0.001 |
| **MI** |  |  |  |  |  |  |
| NMC (reference) | 1 |  | 1 |  | 1 |  |
| HMB | 1.14 (0.74, 1.76) | 0.544 | 1.37 (0.93, 2.00) | 0.111 | 1.73 (1.06, 2.81) | 0.027 |
| **DM** |  |  |  |  |  |  |
| NMC (reference) | 1 |  | 1 |  | 1 |  |
| HMB | 1.29 (1.16, 1.43) | <0.001 | 1.27 (1.15, 1.41) | <0.001 | 1.37 (1.20, 1.55) | <0.001 |

Abbreviations: OR, Odds ratio; CI, Confidence interval; CVD, Cardiovascular disease; MACE, Major adverse cardiovascular event; CHD, coronary heart disease; CVA, Cerebrovascular accident; HF, Heart failure; AF, Atrial fibrillation; MI, Myocardial infarction; DM, Diabetes mellitus; NMC, Normal menstrual cycle; HMB, Heavy menstrual bleeding.

MACE was defined as the composite of myocardial infarction, stroke, and heart failure.

* Adjusted model included age, race/ethnicity, household income quartile, primary payer, smoking, alcohol, contraceptive use, metabolic syndrome, non-steroidal anti-inflammatory drugs, and leiomyoma uterus after excluding anticoagulant cases.

** Adjusted model included age, race/ethnicity, household income quartile, primary payer, smoking, alcohol, contraceptive use, metabolic syndrome, non-steroidal anti-inflammatory drugs, and leiomyoma uterus after excluding NSAID cases.

***Adjusted model included age, race/ethnicity, household income quartile, primary payer, contraceptive use, metabolic syndrome, NSAIDs, and leiomyoma uterus after excluding smoking/alcohol use and obesity.

**Supplementary Table 7. Adjusted direct and indirect associations of HMB diagnosis with CVD outcome through mediators among hospitalizations of women aged ≤40 years using survey-weighted logistic regression analyses**

| **Major adverse cardiovascular events (MACE)** | **Mediation effect** | |
| --- | --- | --- |
|  | **OR* (95% CI)** | **p-value** |
| **Metabolic Syndrome** |  |  |
| Total association of HMB | 1.52 (1.33, 1.74) | <0.001 |
| Direct association of HMB | 1.45 (1.27, 1.66) | <0.001 |
| Indirect effect of HMB via MS | 1.05 (1.04, 1.06) | <0.001 |
| **Obesity** |  |  |
| Total association of HMB | 1.55 (1.31, 1.84) | <0.001 |
| Direct association of HMB | 1.41 (1.18, 1.67) | <0.001 |
| Indirect association of HMB via obesity | 1.10 (1.09, 1.11) | <0.001 |
| **Hypertension** |  |  |
| Total association of HMB | 1.56 (1.33, 1.84) | <0.001 |
| Direct association of HMB | 1.37 (1.17, 1.62) | <0.001 |
| Indirect association of HMB via hypertension | 1.14 (1.12, 1.15) | <0.001 |
| **Diabetes** |  |  |
| Total association of HMB | 1.60 (1.35, 1.89) | <0.001 |
| Direct association of HMB | 1.46 (1.23, 1.74) | <0.001 |
| Indirect association of HMB via diabetes | 1.09 (1.08, 1.11) | <0.001 |
| **Anemia** |  |  |
| Total association of HMB | 1.50 (1.27, 1.78) | <0.001 |
| Direct association of HMB | 1.47 (1.24, 1.74) | <0.001 |
| Indirect association of HMB via anemia | 1.02 (1.02, 1.03) | <0.001 |

Abbreviations: OR, Odds ratio; CI, Confidence interval; MACE, Major adverse cardiovascular event; HMB, Heavy menstrual bleeding; MS, Metabolic Syndrome. MACE was defined as the composite of myocardial infarction, stroke, and heart failure.

*Adjusted model included age, race/ethnicity, household income quartile, primary payer, smoking, alcohol, contraceptive use, non-steroidal anti-inflammatory drugs, and leiomyoma uterus.

**Supplementary Table 8. Interaction between lifestyle factors and HMB on CVD outcomes among hospitalized women of ages 18-40 years using survey-weighted logistic regression analyses**

| **Interaction** | **MACE** | **CHD** | **Stroke /CVA** | **HF** | **AF/ Arrhythmia** | **MI** | **DM** |
| --- | --- | --- | --- | --- | --- | --- | --- |
|  | **p-value** | **p-value** | **p-value** | **p-value** | **p-value** | **p-value** | **p-value** |
| HMB with Smoking/Alcohol | 0.770 | 0.598 | 0.919 | 0.693 | 0.094 | 0.768 | 0.362 |
| HMB with Obesity | 0.170 | 0.274 | 0.874 | 0.226 | 0.036 | 0.223 | 0.003 |
| HMB with MS | 0.024 | 0.057 | 0.399 | 0.024 | 0.279 | 0.596 | 0.721 |

Abbreviations: OR, Odds ratio; CI, Confidence interval; MACE, Major adverse cardiovascular event; HMB, Heavy menstrual bleeding; MS, Metabolic Syndrome. MACE was defined as the composite of myocardial infarction, stroke, and heart failure.

Regression models were adjusted for age, race/ethnicity, household income quartile, primary payer, smoking, alcohol, contraceptive use, metabolic syndrome, non-steroidal anti-inflammatory drugs, and leiomyoma uterus.

**Supplementary Table 9. Adjusted factors associated with MACE outcome among hospitalized women of ages between 18-40 years using survey-weighted logistic regression analyses**

| **Major adverse cardiovascular events** | **OR (95% CI)** | **p-value** |
| --- | --- | --- |
| **Heavy menstrual bleeding** | 1.30 (1.10, 1.54) | 0.003 |
| **Age** (years) | 1.11 (1.11, 1.12) | <0.001 |
| **Race/Ethnicity** |  |  |
| White (reference) | 1 |  |
| Black | 2.05 (1.94, 2.16) | <0.001 |
| Hispanic | 0.79 (0.73, 0.86) | <0.001 |
| Others/Missing | 1.03 (0.93, 1.13) | 0.591 |
| **HH income quartile** |  |  |
| 1st (reference) | 1 |  |
| 2nd | 0.84 (0.80, 0.88) | <0.001 |
| 3rd | 0.72 (0.68, 0.77) | <0.001 |
| 4th | 0.58 (0.54, 0.63) | <0.001 |
| Missing | 0.98 (0.84, 1.14) | 0.750 |
| **Primary payer** |  |  |
| Medicare (reference) | 1 |  |
| Medicaid | 0.37 (0.35, 0.40) | <0.001 |
| Private insurance | 0.21 (0.19, 0.22) | <0.001 |
| Self-Pay | 0.51 (0.47, 0.56) | <0.001 |
| No charge | 0.44 (0.33, 0.59) | <0.001 |
| Other | 0.32 (0.28, 0.36) | <0.001 |
| **Smoking use** | 1.65 (1.42, 1.91) | <0.001 |
| **Alcohol use** | 1.11 (1.00, 1.23) | 0.050 |
| **Contraceptive/Hormone use** | 1.85 (1.46, 2.35) | <0.001 |
| **Metabolic syndrome** | 1.80 (1.66, 1.96) | <0.001 |
| **Obesity** | 1.84 (1.76, 1.93) | <0.001 |
| **Insulin use** | 2.53 (2.37, 2.71) | <0.001 |
| **Polycystic ovary syndrome** | 0.83 (0.71, 0.98) | 0.025 |
| **Anemia** | 1.30 (1.22, 1.37) | <0.001 |
| **Anticoagulant** **use** | 5.31 (4.91, 5.74) | <0.001 |
| **Non-steroidal anti-inflammatory drug use** | 1.04 (0.82, 1.32) | 0.742 |
| **Leiomyoma uterus** | 0.31 (0.25, 0.37) | <0.001 |
| **Inflammatory bowel disease** | 0.92 (0.76, 1.11) | 0.368 |
| **Infertility** | 0.51 (0.19, 1.38) | 0.183 |

Abbreviations: NIS, National inpatient sample; SD, Standard deviation; HH, Household; OR, Odds ratio; CI, Confidence interval. MACE was defined as the composite of myocardial infarction, stroke, and heart failure.

**Supplementary Table 10. Adjusted association of HMB categories with CVD events including diabetes among hospitalized women of ages between 18-40 years using survey-weighted logistic regression analyses**

|  | **Adjusted model* (N=1139431)** | | **Adjusted model** (N=1139431)** | |
| --- | --- | --- | --- | --- |
|  | **OR (95% CI)** | **p-value** | **OR (95% CI)** | **p-value** |
| **MACE** |  |  |  |  |
| NMC (reference) | 1 |  | 1 |  |
| Only HMB | 1.51 (1.26, 1.81) | <0.001 | 1.50 (1.25, 1.79) | <0.001 |
| HMB with IM | 1.2 (0.85, 1.71) | 0.299 | 1.26 (0.88, 1.79) | 0.207 |
| **CHD** |  |  |  |  |
| NMC (reference) | 1 |  | 1 |  |
| Only HMB | 1.32 (1.03, 1.71) | 0.029 | 1.30 (1.01, 1.68) | 0.039 |
| HMB with IM | 1.65 (1.08, 2.52) | 0.020 | 1.74 (1.13, 2.68) | 0.012 |
| **Stroke /CVA** |  |  |  |  |
| NMC (reference) | 1 |  | 1 |  |
| Only HMB | 1.32 (0.9, 1.94) | 0.151 | 1.32 (0.9, 1.93) | 0.156 |
| HMB with IM | 1.34 (0.63, 2.86) | 0.445 | 1.34 (0.63, 2.86) | 0.445 |
| **HF** |  |  |  |  |
| NMC (reference) | 1 |  | 1 |  |
| Only HMB | 1.52 (1.23, 1.87) | <0.001 | 1.50 (1.22, 1.86) | <0.001 |
| HMB with IM | 1.21 (0.81, 1.82) | 0.346 | 1.30 (0.86, 1.95) | 0.210 |
| **AF/ Arrhythmia** |  |  |  |  |
| NMC (reference) | 1 |  | 1 |  |
| Only HMB | 1.89 (1.51, 2.36) | <0.001 | 1.87 (1.5, 2.34) | <0.001 |
| HMB with IM | 1.92 (1.3, 2.86) | 0.001 | 1.94 (1.3, 2.88) | 0.001 |
| **MI** |  |  |  |  |
| NMC (reference) | 1 |  | 1 |  |
| Only HMB | 1.41 (0.93, 2.14) | 0.101 | 1.40 (0.93, 2.11) | 0.108 |
| HMB with IM | 1.02 (0.38, 2.75) | 0.970 | 1.03 (0.38, 2.8) | 0.952 |
| **DM** |  |  |  |  |
| NMC (reference) | 1 |  |  |  |
| Only HMB | 1.31 (1.17, 1.47) | <0.001 |  |  |
| HMB with IM | 1.12 (0.9, 1.39) | 0.295 |  |  |

Abbreviations: OR, Odds ratio; CI, Confidence interval; CVD, Cardiovascular disease; MACE, Major adverse cardiovascular event; CHD, coronary heart disease; CVA, Cerebrovascular accident; HF, Heart failure; AF, Atrial fibrillation; MI, Myocardial infarction; DM, Diabetes mellitus; NMC, Normal menstrual cycle; HMB, Heavy menstrual bleeding; IM, Irregular mensuration.

MACE was defined as the composite of myocardial infarction, stroke, and heart failure.

*Adjusted model included age, race/ethnicity, household income quartile, primary payer, smoking, alcohol, contraceptive use, metabolic syndrome, non-steroidal anti-inflammatory drugs, and leiomyoma uterus.

**Adjusted model included age, race/ethnicity, household income quartile, primary payer, smoking, alcohol, contraceptive use, metabolic syndrome, non-steroidal anti-inflammatory drugs, leiomyoma uterus, and DM.

**Supplementary Table 11. Unadjusted and adjusted associations of HMB with CVD outcomes including diabetes among hospitalized women of age >40 years**

|  | **Unadjusted model**  **(N=** **1291420)** | | **PS-Model***  **(N=22154)** | |
| --- | --- | --- | --- | --- |
|  | **OR (95% CI)** | **p-value** | **OR (95% CI)** | **p-value** |
| **MACE** |  |  |  |  |
| NMC (reference) | 1 |  | 1 |  |
| HMB | 0.23 (0.21, 0.25) | <0.001 | 1.19 (1.01, 1.40) | 0.032 |
| **CHD** |  |  |  |  |
| NMC (reference) | 1 |  | 1 |  |
| HMB | 0.19 (0.17, 0.21) | <0.001 | 1.16 (0.96, 1.40) | 0.120 |
| **Stroke /CVA** |  |  |  |  |
| NMC (reference) | 1 |  | 1 |  |
| HMB | 0.27 (0.21, 0.33) | <0.001 | 0.97 (0.64, 1.47) | 0.890 |
| **HF** |  |  |  |  |
| NMC (reference) | 1 |  | 1 |  |
| HMB | 0.22 (0.2, 0.25) | <0.001 | 1.19 (1.01, 1.40) | 0.040 |
| **AF/ Arrhythmia** |  |  |  |  |
| NMC (reference) | 1 |  | 1 |  |
| HMB | 0.18 (0.16, 0.2) | <0.001 | 0.94 (0.77, 1.15) | 0.552 |
| **MI** |  |  |  |  |
| NMC (reference) | 1 |  | 1 |  |
| HMB | 0.3 (0.25, 0.37) | <0.001 | 1.46 (1.04, 2.04) | 0.027 |
| **DM** |  |  |  |  |
| NMC (reference) | 1 |  | 1 |  |
| HMB | 0.32 (0.3, 0.33) | <0.001 | 1.10 (0.99, 1.21) | 0.066 |

Abbreviations: OR, Odds ratio; CI, Confidence interval; CVD, Cardiovascular disease; MACE, Major adverse cardiovascular event; CHD, coronary heart disease; CVA, Cerebrovascular accident; HF, Heart failure; AF, Atrial fibrillation; MI, Myocardial infarction; DM, Diabetes mellitus; PCOS, Polycystic ovary syndrome; NMC, Normal menstrual cycle; HMB, Heavy menstrual bleeding. MACE was defined as the composite of myocardial infarction, stroke, and heart failure.

The adjusted associations were carried out using propensity scores-matched analysis. The propensity scores model included race/ethnicity, household income quartile, primary payer, smoking, alcohol, contraceptive use, metabolic syndrome, non-steroidal anti-inflammatory drugs, leiomyoma uterus, obesity, PCOS, anemia, and anticoagulants. *Model additionally adjusted for age.

**Supplementary Table 12. Adjusted associations of HMB with CVD outcomes including diabetes among hospitalized women according to age group using propensity scores-matched analyses**

|  | **PS-Model* (Age group: 41 -55)**  **(N=22154)** | | **PS-Model* (Age group: 56 -70)**  **(N=22154)** | |
| --- | --- | --- | --- | --- |
|  | **OR (95% CI)** | **p-value** | **OR (95% CI)** | **p-value** |
| **MACE** |  |  |  |  |
| NMC (reference) | 1 |  | 1 |  |
| HMB | 0.91 (0.56, 1.48) | 0.692 | 1.24 (1.05, 1.47) | 0.013 |
| **CHD** |  |  |  |  |
| NMC (reference) | 1 |  | 1 |  |
| HMB | 1.01 (0.58, 1.76) | 0.973 | 1.18 (0.97, 1.43) | 0.092 |
| **Stroke /CVA** |  |  |  |  |
| NMC (reference) | 1 |  | 1 |  |
| HMB | 0.60 (0.14, 2.50) | 0.484 | 1.03 (0.66, 1.58) | 0.912 |
| **HF** |  |  |  |  |
| NMC (reference) | 1 |  | 1 |  |
| HMB | 1.06 (0.64, 1.75) | 0.824 | 1.24 (1.04, 1.48) | 0.018 |
| **AF/ Arrhythmia** |  |  |  |  |
| NMC (reference) | 1 |  | 1 |  |
| HMB | 1.96 (1.24, 3.09) | 0.004 | 0.87 (0.70, 1.08) | 0.203 |
| **MI** |  |  |  |  |
| NMC (reference) | 1 |  | 1 |  |
| HMB | 1.35 (0.42, 4.31) | 0.614 | 1.36 (0.97, 1.91) | 0.075 |
| **DM** |  |  |  |  |
| NMC (reference) | 1 |  | 1 |  |
| HMB | 1.06 (0.74, 1.51) | 0.749 | 1.12 (1.01, 1.24) | 0.030 |

Abbreviations: OR, Odds ratio; CI, Confidence interval; CVD, Cardiovascular disease; MACE, Major adverse cardiovascular event; CHD, coronary heart disease; CVA, Cerebrovascular accident; HF, Heart failure; AF, Atrial fibrillation; MI, Myocardial infarction; DM, Diabetes mellitus; NMC, Normal menstrual cycle; HMB, Heavy menstrual bleeding. MACE was defined as the composite of myocardial infarction, stroke, and heart failure. The adjusted associations were carried out using propensity scores-matched analysis. The propensity scores model included race/ethnicity, household income quartile, primary payer, smoking, alcohol, contraceptive use, metabolic syndrome, non-steroidal anti-inflammatory drugs, leiomyoma uterus, obesity, PCOS, anemia, and anticoagulants. *Model additionally adjusted for age.
